# Fast Liquid Chromatography Coupled with Tandem Mass Spectrometry for the Analysis of Vanillic and Syringic Acids in Ice Cores

**DOI:** 10.1021/acs.analchem.1c05412

**Published:** 2022-03-23

**Authors:** Elena Barbaro, Matteo Feltracco, Azzurra Spagnesi, Federico Dallo, Jacopo Gabrieli, Fabrizio De Blasi, Daniele Zannoni, Warren R.L. Cairns, Andrea Gambaro, Carlo Barbante

**Affiliations:** † Institute of Polar Sciences, National Research Council (CNR-ISP), Via Torino, Venice Mestre (VE) 155-30172, Italy; ‡ Department of Environmental Sciences, Informatics and Statistics, Ca’ Foscari University of Venice, Via Torino, Venice Mestre (VE) 155-30172, Italy; § Center for the Built Environment, University of California, 390 Wurster Hall #1839, Berkeley, California 94720-1839, United States; ∥ Geophysical Institute, University of Bergen and Bjerknes Centre for Climate Research, Postboks 7803, Bergen NO-5020, Norway

## Abstract

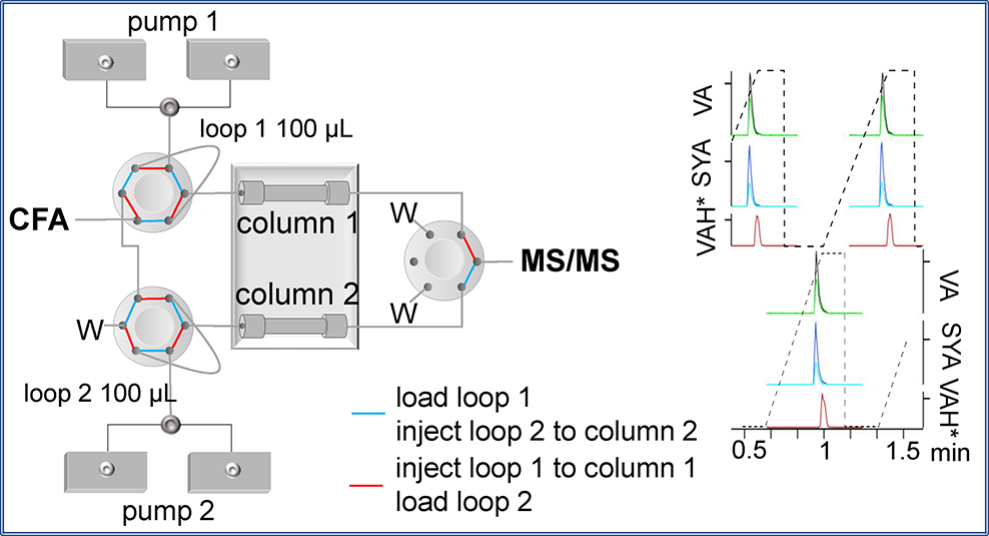

The development of new analytical systems and the improvement of the existing ones to obtain high-resolution measurements of chemical markers in samples from ice cores, is one of the main challenges the paleoclimatic scientific community is facing. Different chemical species can be used as markers for tracking emission sources or specific environmental processes. Although some markers, such as methane sulfonic acid (a proxy of marine productivity), are commonly used, there is a lack of data on other organic tracers in ice cores, making their continuous analysis analytically challenging. Here, we present an innovative combination of fast liquid chromatography coupled with tandem mass spectrometry (FLC-MS/MS) to continuously determine organic markers in ice cores. After specific optimization, this approach was applied to the quantification of vanillic and syringic acids, two specific markers for biomass burning. Using the validated method, detection limits of 3.6 and 4.6 pg mL^–1^ for vanillic and syringic acids, respectively, were achieved. Thanks to the coupling of FLC-MS/MS with the continuous flow analytical system, we obtained one measurement every 30 s, which corresponds to a sampling resolution of a sample every 1.5 cm with a melting rate of 3.0 cm min^–1^. To check the robustness of the method, we analyzed two parallel sticks of an alpine ice core over more than 5 h. Vanillic acid was found with concentrations in the range of picograms per milliliter, suggesting the combustion of coniferous trees, which are found throughout the Italian Alps.

## Introduction

Ice cores are excellent archives of past atmospheric composition since valuable paleoclimatic information can be obtained from chemical analysis of the dissolved and particulate matter trapped within the ice. Impurities are progressively accumulated in the ice layers through wet or dry deposition of aerosol or by direct trapping in the gas phase. During transport, or after deposition, some compounds can even undergo photochemical reactions that produce more stable chemical species.

A flourishing section of the paleoclimatic literature is reserved for the determination of specific inorganic impurities in ice cores (e.g., insoluble dust particles, black carbon, sulfates and nitrates, major ions, trace elements, and isotopes of some elements) since they are tracers for emission sources or markers for some environmental processes.^1−4^ Inorganic primary marine aerosol represents one of the most studied classes of components in ice. Sodium, in particular, is normally used to characterize the sea salt inputs after mathematical correction for terrestrial contributions.^5^ Special attention has also been paid to insoluble dust particles and some elemental markers for terrestrial compounds derived from soils such as Al, Fe, or Ca.^2,6^

There is growing interest in discovering and determining tracers for organic marine aerosols. Although organic compounds make up a large proportion of atmospheric aerosol,^7^ their investigation in ice cores is limited to methanesulfonic acid,^8−10^ perchlorate,^11^ short-chain carboxylic acids,^12−14^ and some markers for biomass burning such as levoglucosan.^15−20^ Most tracers for biomass burning can have multiple sources (e.g., ammonium, formate, acetate, potassium, and nitrate) and so are less specific than the combustion products of cellulose such as levoglucosan. However, this class of compounds does not allow specific differentiation between the type of vegetation burnt during biomass burning events.

Although their use is still limited in the literature, phenolic compounds, such as vanillic (VA) and syringic acids (SYA), are particularly useful in the systematic reconstruction of fire history and are crucial markers that help identify source combustion areas. Their origin from lignin combustion makes them key indicators of the type of vegetation burnt, helping in the distinction between conifers and deciduous tree species.^21−23^

Determining the organic compound load of polar and alpine ice is an analytical challenge because single organic compounds are present at trace and ultra-trace concentrations; in addition, there is a lack of knowledge on their post-depositional behavior in ice, including possible degradation processes.

In the last few years, the ice core and environmental archive scientific community has focused on the development and improvement of new analytical systems to obtain measurements of chemical markers in samples from environmental archives with a much higher temporal resolution than previously possible. Particular attention has been paid to the reconstruction of the chemical stratigraphy from deep drilled ice cores from polar regions.^5,24^ The newest challenge will be once the “Beyond EPICA—Oldest Ice Core” (BE-OIC) project, recently funded by European Community, is completed. The aim of this project is to understand why the climate change frequency shifted from 40 kyr cycles to 100 kyr cycles, starting from the Mid–Pleistocene Transition, about 1 Myr ago. The analytical methods being developed will have to resolve the chemical composition of the compressed ice layers to achieve the highest temporal resolution possible with highly reliable throughput as the core can only be melted once. Recent advances in analytical technology have been expected to be of great help in obtaining new climatic information from newly identified environmental and climatic chemical markers.

Since the early 90s, sample preparation used for ice core analysis has evolved from discrete samples to continuous melting, allowing higher temporal and spatial resolution, with the additional advantage of reduced contamination as sample handling has been minimized. Typical preparation of discrete samples consisted of the manual removal of externally contaminated ice layers with a pre-cleaned ceramic knife^25^ or by washing the outermost section three times using ultrapure water.^6^ The application of such pre-analytical methods was and remains time-consuming, especially when considering the large number of samples that need to be handled, exposing the samples to possible contamination, damage, or loss.

Techniques for the continuous melting of ice, collectively known as continuous flow analysis (CFA), rapidly developed from the early 1990s onward. From the original design at the University of Bern,^26^ CFA is now applied to continuous measurements of physical and chemical parameters such as soluble and insoluble impurities, air gases, water stable isotopes, acidity, and conductivity in ice cores. Along with increased applicability, the systems have become more compact, modular, robust, and transportable. Contamination of the samples has been minimized by using differential pump rates to separate the outer more contaminated meltwater from the inner cleaner meltwater flow, ensuring an efficient decontamination.^27^ The outer layer meltwaters are potentially more contaminated as they have been in contact with the drill surface and drilling fluids. Instead, contamination risk of the inner core is reduced by using high-purity melt-head materials and/or coatings to protect the sample.^27^

Until now, CFA has mainly been coupled with analytical techniques that allow continuous sample analysis, such as flow injection analysis,^28−30^ or inductively coupled plasma–mass spectrometry (ICP–MS).^27,31,32^ Traversi and her colleagues^33^ have developed a way of coupling fast ion chromatography (FIC) to a melter system for quantitative continuous determination of major ionic chemical impurities in ice. This has been improved over the last decade by improving the temporal resolution of the analyses and the chromatographic performance.^34−37^

This paper proposes the coupling of fast liquid chromatography–tandem mass spectrometry (FLC-MS/MS) with a CFA system to continuously determine organic compounds in ice cores. Although similar to FIC, this instrumental combination has never been used before. The advantage of FLC-MS/MS is that it can be used to determine any organic compound after optimization of the chromatographic and mass spectrometric parameters, while FIC is limited to the determination of ions.

We here propose the first CFA-FLC-MS/MS method for the determination of VA and SYAs; these two phenolic compounds are biomass burning tracers that have been widely determined in different environmental matrices, including ice cores.^17−19^ However, they have never been determined continuously from an ice core to preserve their high temporal resolution in the sample. Although the key tracer for biomass burning is levoglucosan,^38^ phenolic compounds such as VA and SYAs can be used to differentiate between the types of vegetation burned.^39^ Softwood during combustion principally generates compounds with vanillyl moieties,^21^ lignin in hardwoods produces syringil and vanillyl moieties,^23^ while grass combustion results in coumaryl moieties.^40^ The FLC-MS/MS method developed has been applied to a shallow ice core section collected from the summit plateau of the Corbassiere glacier in the saddle between Grand Combin de Grafeneire and Grand Combin de Valsorey (Switzerland, 4100 m asl). The ice core was collected during the ICE MEMORY project expedition in September 2020.

## Experimental Section

**Figure 1:**
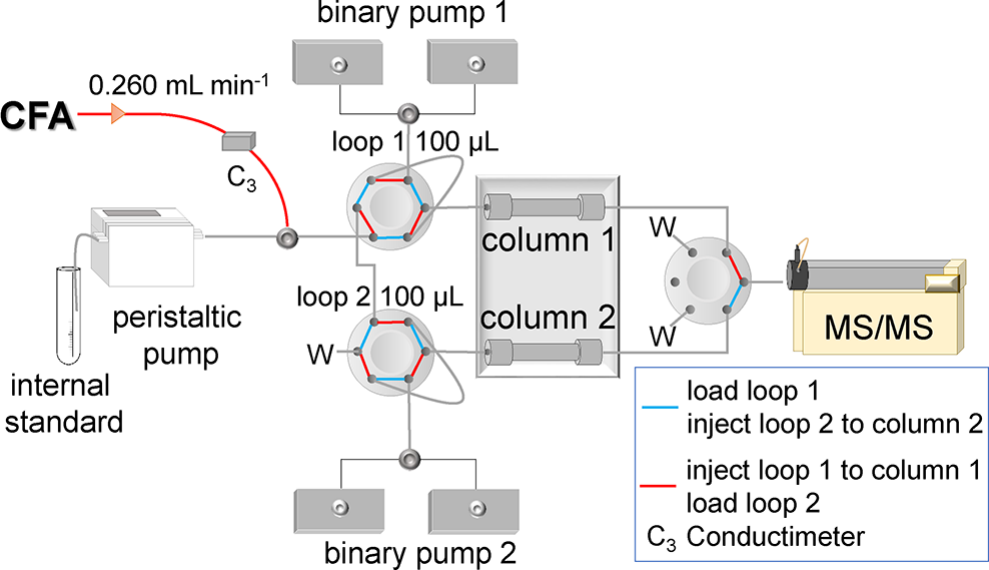
Scheme of FLC-MS/MS.

The challenge when developing the FLC-MS/MS setup was to obtain a very fast chromatographic run that minimized the amount of unutilized meltwater and reduced dispersion to increase the vertical resolution of the analysis of the ice core. This resolution does not depend only on the analytical sampling rate of the meltwater streams, as during discrete sampling with a fraction collector. It also depends on the dispersion of the analyte concentrations in the meltwater.^34^ Resolution can be enhanced by decreasing the melt rate, but variations in the flow rate of the meltwater stream, and also in the total system void volume, can affect the dispersion of the analytical signals and therefore the actual sampling resolution.^34^ For this reason, particular attention was paid to adjusting a specific chromatographic system to allow coupling with CFA. Figure 1 shows a schematic representation of the FLC-MS/MS system, while the details of the CFA system are reported in the Supporting Information and Figure S1. The melting system was modified with a dedicated flow line for the FLC-MS/MS instrument. We used a completely scalable home-made software written in LabView for managing and controlling the whole CFA system (the parameters included melting speed rate and ice column height). Further modifications were made to guarantee a constant, minimal, and mostly air bubble-free sample flow by installing an inject-load switching valve and two debubblers. These modifications improved the system robustness and made our approach particularly suitable for coupling with FLC-MS/MS. This method allows for reliable continuous measurements. Other analytical techniques for organic compounds in ice core sub samples were typically based on discrete samples obtained by cutting the ice and melting it or using a continuous melting system with fraction collection, resulting in a lower spatial resolution sampling of the ice core.^33^ Our system with these upgrades can be potentially coupled with other analytical instruments, given that there are currently unused lines leaving the five-port sampling manifold.

### Chromatographic System

A dedicated peristaltic pump was used to continuously load the meltwater into the FLC system at a flow rate of 260 ± 14 μL min^–1^(*n* = 5). This flow is optimized to completely fill the loops, but in the future, it can be adjusted to minimize the overflow, thus reducing the amount of unused meltwater. An internal standard (IS) solution is continuously added to the sample flow through a T-connector connected to the third peristaltic pump equipped with Tygon tubing operating at a flow rate of 14.5 ± 0.2 μL min^–1^(*n* = 5). The IS is isotopically labeled vanillin ^13^C_6_ (VAH*) from Sigma-Aldrich. The low IS flow minimizes the sample dilution to 6% and minimized the contamination risk despite the use of Tygon tubing. Tygon was used because no FPM tubing below 0.38 mm ID was commercially available. Quantification using an IS was used as it can correct random fluctuations, isotopically labeled VAH* was chosen as it has a similar chromatographic behavior and ionization in the mass spectrometer, to the target analytes. The IS is transported in ultrapure water, and no significant blank contribution was found.

**Figure 2:**
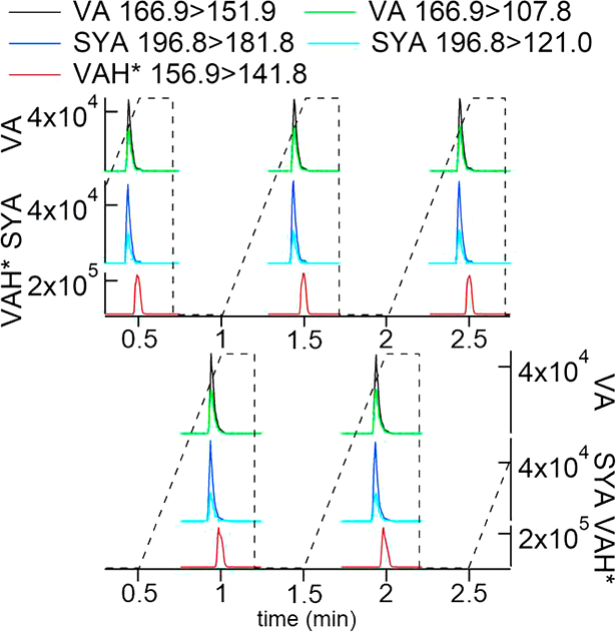
Extracted ion chromatogram for each MRM transition of VA, SYAs, and IS-labeled vanillin (VAH*) at a concentration of 1 ng mL^–1^. Dashed lines show the % of eluent B used in the chromatographic gradient. The cleaning and the equilibration steps start at 30 s.

The chromatographic system used is the Thermo Scientific Dionex UltiMate 3000 Rapid Separation LC system, consisting of two separate dual-gradient Rapid Separation Pumps (DGP-3600RS, Thermo Fisher Scientific). These are used to obtain the fastest run times possible. These pumps are equipped with 10 μL mixers with an innovative SpinFlow mixing design for high mixing performance with a low gradient delay for fast separations. A column oven (DGP-3600RS, Thermo Fisher Scientific) is used to maintain the two 2.1 × 30 mm Acclaim 120 C18 Columns (particle size 2.2 μm, Thermo Fisher Scientific) at 40 °C. Two 6-Port Valves are used to load and inject the samples every 30 s. When the first loop (100 μL) is in the inject position (red lines in Figure 1), elution of the analytes occurs in the first column and the divert valve sends the eluent to the MS. While this is happening, the second loop (100 μL) is loading with sample stream from the melter. The two valves are connected with peek tubing with a volume of 5 μL. After 30 s, the two injection valves and the divert valve are switched, and separation in column 2 occurs while loop 1 is loading and column 1 is cleaning and equilibrating (Figure 1). These two steps cycle repeatedly for the duration of the melting session. The analytes were eluted using a linear gradient with HiPerSolv Chromanorm water for HPLC (VWR) with 0.01% v/v of formic acid (LC–MS LiChropur by Sigma-Aldrich) as eluent A and HiPerSolv Chromanorm acetonitrile (VWR) as eluent B. The binary elution program for the whole system is as follows and is also reported as the dashed line in Figure 2.

With the column flow rates set at 0.5 mL min^–1^, the elution program of column 1 is as follows: 0 min, 40% eluent B; 0–0.5 min gradient from 40 to 100%, 0.5–0.7 min, 100% eluent B (cleaning); and 0.7–1 min, equilibration with 40% eluent B. The binary elution program of the second column is the same but with a time delay of 0.5 min: so, 0.5 min, 40% eluent B; 0.5–1.0 min gradient from 40% to 100%, 1–1.2 min, 100% eluent B (cleaning); and 1.2–1.5 min, equilibration with 40% eluent B. The elution programs were cycled repeatedly to obtain a final run time of 40 min, the time required to analyze a 70 cm ice core section.

The divert valve switches alternatively to send to the mass spectrometer the flow coming from either the first column (i.e., 0–0.5 min) or the second column (i.e., 0.5–1 min).

### Mass Spectrometer

The chromatographic system is coupled with an API 4000 Triple Quadrupole Mass Spectrometer (Applied Biosystem/MSD SCIEX, Concord, Ontario, Canada) using a Turbo V electrospray source (ESI) that operated in the negative ion mode. Data were collected in the multiple reaction monitoring (MRM) mode. The first quadrupole (Q1) selected the molecular ion, while the third quadrupole (Q3) selected the fragment. Both Q1 and Q3 were set at unit resolution with a peak width of 0.7 ± 0.1 amu at 50% of maximum peak height. To improve the sensitivity, declustering potential (DP), cell energy (CE), and cell exit potential (CXP) were optimized using direct infusions of 1 mg L^–1^ of each individual standard in order to find the best parameters to maximize the mass spectrometer signal. The voltage of the orifice was controlled by the DP parameter, the CE was the amount of energy that the precursor ions received as they were accelerated into the collision cell, and the CXP was used to focus and accelerate the ions after leaving the collision cell. The monitored transition and the analyzer parameters for VA, SyA, and vanillin (VAH*) are summarized in Table S2, and their optimization was performed as in our previous paper.^46^ Due to a different chromatographic setup being used, gas flows and the source temperature were optimized by injection of a 1 ng mL^–1^ standard solution. The source’s parameters were set as follows: temperature 650 °C, ion spray voltage −4450 V, GS1 45 psi, GS2 60 psi, and CUR 30 psi.

### Ice Core Samples

In this work, the deepest section of the Grand Combin shallow core, from 24.35 to 25.10 m of depth, has been analyzed. From the whole body of the ice core (7.5 cm diameter), we were able to obtain two parallel sections (3.2 × 3.2 × 70 cm) that were used to evaluate the method reproducibility. The ice has been cut with a modified commercial band saw with a decontaminated stainless-steel blade on a polyethylene tabletop with polyethylene guides. The table, guides, and the blade were carefully cleaned with acetone and methanol to remove contamination before use. All exposed ice surfaces were rapidly scraped with a stainless steel knife previously cleaned with 0.1% v/v ultra-pure HNO_3_ (Romil, Cambridge, UK), then rinsed several times, and carefully dried after each use. This knife was used to remove the thin outer contaminated ice layer. The end faces were then scraped again using a second clean knife that removed several millimeters of ice from each end. The sections have been stored in clean poly(tetrafluoroethylene) bags until analysis. Before analysis, each sample was inserted in a polyethylene holder and placed on the melting head. Two 50 mm long pieces of frozen ultra-pure water (Purelab Ultra-Analytic, Elga LabWater, High Wycombe, UK) were employed to determine the blank levels and provide a baseline before and after each melting procedure. To prevent contamination from the outer layers of the core sections, the melting head surface geometry consists of two concentric areas that divide the sample flows so that only the inner part of the samples (2.1 × 2.1 cm, ∼46%) is used for analysis, while the external section of the ice is discarded or used for analyses that suffer less from contamination.

## Results and Discussion

### Instrumental Performance

Figure 2 shows the extracted chromatograms for each transition monitored using the present method. The figure shows a 5 min representative run, but the cyclic sequence can be extended for any length of time. The retention times of SyA, VA, and VAH* are 20.4, 22.2, and 24.6 s, respectively. Chromatographic resolution between each species is not necessary when a mass spectrometer is used because it allows separation of species with the same retention time based on their *m*/*z*. This assumption only holds if interferences are absent for each specific transition. For this reason, a preliminary check of possible interference is evaluated using ultrapure water as a procedural blank. However, chromatographic separation prior to mass spectrometric detection is essential to separate the analytes from other compounds and to focus the analyte peaks, thus improving the signal-to-noise ratio.

The MS dwell time, the time spent acquiring the targeted MRM transition during each scan cycle, is a critical parameter because the chromatographic peak width is only a few seconds (Table S3) and a minimum number of identification points is necessary. European Commission decision 2002/657/EC, implementing the Council Directive 96/23/EC concerning the performance of analytical methods and the interpretation of results, establishes a minimum of three or four identification points to identify a targeted analyte. For LC coupled with a tandem mass spectrometer, this is achieved by acquiring two MRM transitions: the most intense transition (highlighted in Table S3 and shown in Figure 2) is used for sample quantification, while another transition is used to confirm compound identity. Using 50 milliseconds as the MS dwell time, the identification points for each compound in the most intense transition are always more than 4, although the peak widths are only 4–5 s (Table S3).

The accordance between column 1 and column 2 is evaluated (Table S3) by considering the peak area, peak width, peak asymmetry, and MS identification points. Both columns can be considered as a unified analytical system because no significant differences are found considering the standard deviation or relative standard deviation. Moreover, we performed the Wilcoxon signed-rank test on the peak area values of SyA and VA because the distribution cannot be assumed to be normally distributed, and this test confirms that the distributions were not significantly different.

Chromatographic performance can also be evaluated using peak asymmetry. This parameter describes the significant deviation of the peak shape from a symmetrical peak shape: when >1, a tailing effect occurs, while <1, fronting is present. The most important reasons for the presence of asymmetry are slow mass transfer, column overload, heterogeneity of the stationary phase surface, and heterogeneity of the column packing.^41^ Each chromatographic peak shows an asymmetry value close to 1 but always above 1 (Table S3) because a slight tailing effect is recorded for every peak (Figure 2).

### Quantitative Performance

Quantitative performance was evaluated in terms of detection and quantification limits, linear range, reproducibility, and matrix effects. Each standard solution was loaded into the injection-load valve located after the melting system but before the first peristaltic pump, so that each solution passes through the entire CFA system (Figure S1).

The instrumental limit of detection (LOD) and quantification (LOQ) limits are defined as 3 and 10 times the signal-to-noise ratio, respectively,^42^ of known absolute amounts of the analyzed target compound in a standard solution. These values are calculated by evaluating the instrumental signal and noise in 10 replicates of the standard solutions at 10 pg mL^–1^. The LOD and LOQ values are found to be 4.8 ± 0.4 and 16 ± 1 pg mL^–1^ for SyA, while the LOD and LOQ values of 2.9 ± 0.3 and 10 ± 1 pg mL^–1^ are found for VA (Table 1). Grieman et al.^43^ determined VA in ice cores using HPLC-MS/MS, obtaining an LOD value (77 pg mL^–1^) 25 times higher than our value (3.6 ± 0.3 pg mL^–1^). This improvement is probably due to (1) the use of a ultrahigh-performance liquid chromatography system with a peak width of only a few seconds compared to the peak widths >0.5 min in the cited method; (2) the higher organic solvent content in the mobile phase during ionization; and (3) the different ionization source in the mass spectrometer. The LOD values of SyA and VA of the present method are slightly lower than 9 and 5 pg mL^–1^, respectively, found by Grieman et al.^18^ with the IC-ESI-MS/MS method. Our LOD values are low enough to determine these compounds in Greenland ice cores because VA levels were found from <LOD to 80 pg mL^–1^ in the Tunu ice core over the past 1700 years.^17^ The VA concentrations ranged from below the LOD to 200 pg mL^–1^ in the Lomonosovfonna ice core^19^ (Svalbard Islands). To the best of our knowledge, no information is available for VA concentrations in Antarctic ice cores. Phenolic compounds, including VA and SyA, were determined in aerosol samples collected at Dome C (Antarctic plateau) during the 2011–2012 and 2012–2013 summer campaigns with median total atmospheric concentrations of 15.0 and 7.3 pg m^–3^, respectively.^44^ Considering that the median aerosol concentration of phenolic compounds in Svalbard^45^ was similar at 19 pg m^–3^, we can suppose that the FLC-MS/MS method can also be applied to determine VA and SyA in Antarctic ice cores.

**Table 1:** Summary of Quantitative Parameters: Instrumental LOD and LOQ, Linear Range Parameters without and with IS, Error Percentage as Deviation from the “True” Value, and Relative Standard Deviation (RSD %) for the Different Concentration Levels

	SyA	VA
LOD (pg mL^–1^)	4.8 (0.4)	3.6 (0.3)
LOQ (pg mL^–1^)	16 (1)	12 (1)
without IS		
slope	97	101
*R*^2^	0.999	0.999
with IS		
slope	0.4	0.4
*R*^2^	0.994	0.995

**pg mL**^**–1**^	**Error %(RSD %)(*n*** = **10)**	
10	–8 (10)	10 (5)
50	–10 (2)	10 (5)
100	–10 (2)	1 (5)
200	–7 (8)	2 (9)
500	–10 (2)	–9 (7)
1000	–4 (7)	–4 (7)
2000	–4 (8)	–4 (7)
5000	1 (8)	1 (7)

The linearity of the calibration curves was checked with and without VAH* as the IS using a series of standard solutions prepared in ultrapure water at concentrations of 0.01, 0.05, 0.1, 0.2, 0.5, 1, 2, and 5 ng mL^–1^. The *R*^2^ values were always above 0.99 (Table 1).

VAH* as IS at a final concentration of 1.5 ng mL^–1^ after dilution was continuously mixed into the sample stream though a T-connector before the injection valves. Considering the concentration ratios of VA/VAH* and SyA/VAH* in the standard solutions and the ratio between the relative peak areas, we have obtained an *R*^2^ > 0.99 (Table 1). The VA and SyA concentrations in the samples were calculated using a response factor calculated as (VA_area_ or SyA_area_/VAH*_area_)·([VAH*]/[VA] or [SyA]) using the calibration solution at 1 ng mL^–1^.

The reproducibility was tested by carrying out 10 replicate measurements of the peak area (VA_area_ or SyA_area_/VAH*_area_) at every concentration level (Table 1). The instrumental precision was evaluated, and the RSD % value was below 10%.

To ensure that our analytical method could be applied to real samples, we needed to evaluate its accuracy, that is, the degree of closeness of the determined value to the known “true” value. It is expressed as an error percentage, calculated as (*Q* – *T*)/*T* × 100, where *Q* is the determined value and *T* is the “true value”. Reference certified materials are not available for VA and SyA in ice cores, so the accuracy was checked by excluding each point in turn from both calibration curves and then quantifying them as a real sample. Table 1 reports that the error percentage was always below ±10%, demonstrating an accurate quantification of each analyte.

Since polar ice cores are complex mixtures of organic and inorganic compounds, we quantified the matrix effect by dividing the signal area of each spiked standard at 1 ng mL^–1^ in a selected discrete sample with negligible VA and SyA concentrations with the area of each standard (1 ng mL^–1^) in ultrapure water.^46^ When no matrix effect is observed, a value of 100% is obtained; a value > 100% indicates ionization enhancement, while a value < 100% is indicative of ionization suppression.^46^ Both VA and SyA demonstrated a weak suppression with a signal recovery of 95 ± 5%, for both of them, while a recovery of 96 ± 2% was obtained for the IS VAH*, demonstrating its suitability and similar behavior in the samples. Looking at the area ratios between SyA or VA with VAH*, the IS successfully corrected these weak matrix effects because a result of 99 ± 2% was found in the ice core sample analyzed in this study. This means that the IS continuously corrects the matrix effects on VA and SyA during the FLC-MS/MS analysis.

### Method Application to an Ice Core Section

The quantification method for VA and SyA using the melting system coupled with FLC-MS/MS was applied to two parallel 70 cm sticks of an ice shallow core in the depth range of 24.35–25.10 m collected at the Grand Combin glacier. The present paper does not aim to interpret the climatic and environmental trends of VA and SyA. Thus, this real sample is used to verify the applicability of the FLC-MS/MS method for the continuous analysis of an ice core.

**Figure 3:**
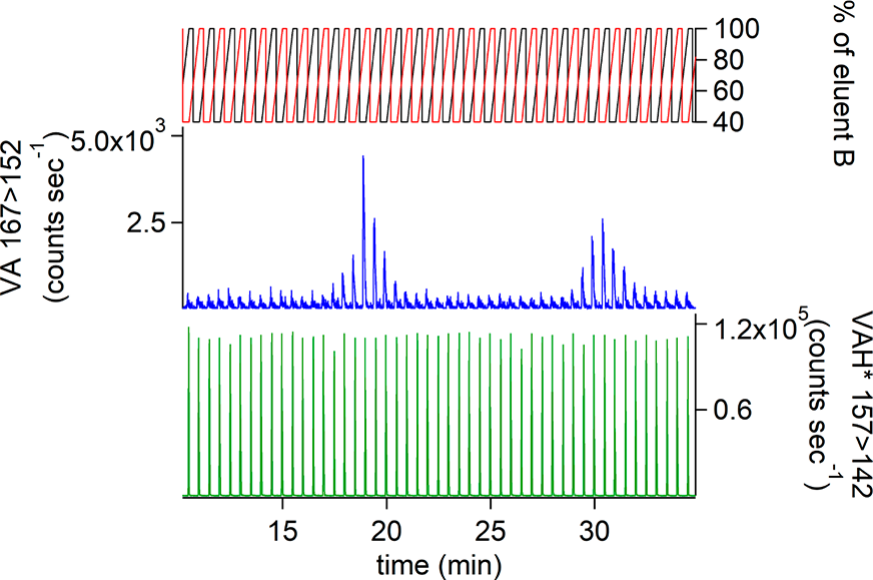
Chromatograms of VA and IS vanillin (VAH*) in the second stick of the Gran Combin ice section. The first panel shows the gradient of eluent B (%) of the mobile phase.

Figure 3 reports the chromatograms relative to the quantitative MRM transitions of VA and IS VAH* during the analysis of the second ice core stick. The use of the IS in the FLC-MS/MS method is the perfect approach for correcting instrumental fluctuations, assuring an accurate quantification for each replicate.

**Figure 4:**
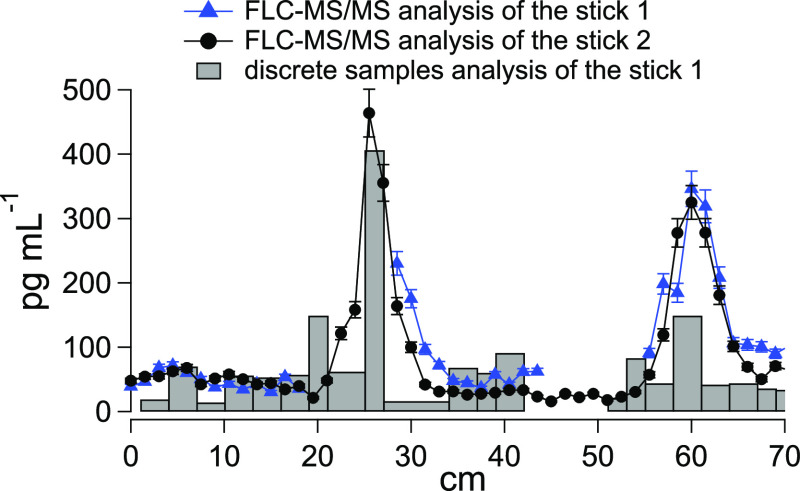
Comparison of the concentrations of VA between two FLC-MS/MS runs for two parallel sticks and the analysis of discrete samples from stick 1 collected using a fraction collector of a 70 cm section of the Gran Combin ice core.

The analysis of both sticks using FLC-MS/MS showed that the results were reproducible (Figure 4). SyA concentrations were below the LOD, while VA was found at concentrations between 30 and 346 pg mL^–1^ for the first stick and between 16 and 464 pg mL^–1^ for the second stick. The presence of VA can probably be explained by the possible combustion of coniferous trees^45^ that are typical to the Italian Alps.^47^

In Figure 4, VA concentration profiles, as a function of depth for both parallel sticks of the same section of Gran Combin ice core, are in excellent agreement even though problems occurred during the analysis of stick 1, resulting in the loss of some data points. Bubble formation during sample melting, and their removal, is one of the critical points of the analysis. Bubbles can cause pressure fluctuations, resulting in unpredictable variations in the chromatographic baseline. This alters the retention time for the analytes and makes peak quantification inaccurate. When bubbles were spotted in the sample line concurrently with a drop in the conductivity signal, the line from the melting system was switched to waste, and the ultrapure water flow was sent to the FLC-MS/MS instrument as detailed in the Experimental Section. The problem was resolved during the run, allowing the acquisition of data from the rest of the sample stick.

Figure 4 also shows a comparison between the concentration values obtained using the FLC-MS/MS method and the analysis of discrete samples of stick 1 collected by the fraction collector. The samples were weighed to obtain a precise volume and were then spiked with the IS. Analysis of the discrete samples was performed using the same method reported in the Experimental Section for FLC-MS/MS analysis by manual injection of the samples into the loops with a syringe.

The profiles show a similar pattern with concentrations in good agreement. What is immediately obvious is the improved depth resolution of the signal with the melting system. The peak at 26 cm is described by three discrete samples or 9 data points directly from the melting system. Although some differences between sticks are to be expected due to heterogeneities in the ice, the discrete and continuous analysis methods showed similar trends (Figure 4). However, the exact reasons for the differences especially at the 60 cm peak are not known, although sample dilution and compound evaporation could contribute. During melting of the first stick, the inject-load valve was switched to ultra-pure water to minimize the ingress of bubbles into the FLC-MS/MS instrument. This ultra-pure water in the system probably contributed to dilution of the samples in the fraction collector. Moreover, volatile carbonaceous compounds, such as methoxyphenols, could evaporate during fraction collection.

## Conclusions

This study presents an innovative approach to determining organic tracers in ice cores in a continuous manner. Here, we present the combination of CFA with FLC-MS/MS. This first method aims to quantify VA and SYAs, specific tracers for biomass burning.

The proposed method shows high sensitivity with detection limits of 3.6 and 4.8 pg mL^–1^ for VA and SYAs, respectively. The limits obtained here were lower than those obtained by discrete methods for ice core analysis. Linear ranges were evaluated between 10 pg mL^–1^ and 5 ng mL^–1^, and *R*^2^ > 0.99 were always obtained. Reproducibility as RSD % was calculated for eight concentration levels and was always below 10%.

The applicability of the FLC-MS/MS system was tested using two parallel sticks of a 70 cm section of an ice core collected from the Gran Combin glacier. The concentration patterns obtained using the FLC-MS/MS system were very similar, while comparison with discrete samples demonstrated some incongruences when experimental problems occurred. Future improvements of the coupling between CFA and FLC-MS/MS will be made to improve the vertical resolution and improve robustness. Particular attention will focus on the debubblers and on the melter head to reduce the melting speed.

## Supporting Information Available

Optimized mass spectrometric parameters for VA and SYAs and _13_C_6_ vanillin as an IS and chromatographic parameters of VA and SYAs to evaluate the instrumental performance (PDF)

## References

[ref1] Buiron D.; Stenni B.; Chappellaz J.; Landais A.; Baumgartner M.; Bonazza M.; Capron E.; Frezzotti M.; Kageyama M.; Lemieux-Dudon B.; Masson-Delmotte V.; Parrenin F.; Schilt A.; Selmo E.; Severi M.; Swingedouw D.; Udisti R. Regional Imprints of Millennial Variability during the MIS 3 Period around Antarctica. Quat. Sci. Rev. 2012, 48, 99–112. 10.1016/j.quascirev.2012.05.023.

[ref2] Caiazzo L.; et al. Prominent Features in Isotopic, Chemical and Dust Stratigraphies from Coastal East Antarctic Ice Sheet (Eastern Wilkes Land). Chemosphere 2017, 176, 273–287. 10.1016/j.chemosphere.2017.02.115. 28273535

[ref3] Eight Glacial Cycles from an Antarctic Ice Core. Nature 2004, 429, 623–628. 10.1038/nature02599. 15190344

[ref4] Stenni B.; et al. Expression of the Bipolar See-Saw in Antarctic Climate Records during the Last Deglaciation. Nat. Geosci. 2011, 4, 46–49. 10.1038/ngeo1026.

[ref5] Wolff E. W.; et al. Changes in Environment over the Last 800,000 Years from Chemical Analysis of the EPICA Dome C Ice Core. Quat. Sci. Rev. 2010, 29, 285–295. 10.1016/j.quascirev.2009.06.013.

[ref6] Ruth U.; Barbante C.; Bigler M.; Delmonte B.; Fischer H.; Gabrielli P.; Gaspari V.; Kaufmann P.; Lambert F.; Maggi V.; Marino F.; Petit J.-R.; Udisti R.; Wagenbach D.; Wegner A.; Wolff E. W. Proxies and Measurement Techniques for Mineral Dust in Antarctic Ice Cores. Environ. Sci. Technol. 2008, 42, 5675–5681. 10.1021/es703078z. 18754492

[ref7] Giorio C.; Kehrwald N.; Barbante C.; Kalberer M.; King A. C. F.; Thomas E. R.; Wolff E. W.; Zennaro P. Prospects for Reconstructing Paleoenvironmental Conditions from Organic Compounds in Polar Snow and Ice. Quat. Sci. Rev. 2018, 183, 1–22. 10.1016/j.quascirev.2018.01.007.

[ref8] Hansson M. E.; Saltzman E. S. The First Greenland Ice Core Record of Methanesulfonate and Sulfate over a Full Glacial Cycle. Geophys. Res. Lett. 1993, 20, 1163–1166. 10.1029/93GL00910.

[ref9] Isaksson E.; Kekonen T.; Moore J.; Mulvaney R. The Methanesulfonic Acid (MSA) Record in a Svalbard Ice Core. Ann. Glaciol. 2005, 42, 345–351. 10.3189/172756405781812637.

[ref10] Saltzman E. S.; Dioumaeva I.; Finley B. D. Glacial/Interglacial Variations in Methanesulfonate (MSA) in the Siple Dome Ice Core, West Antarctica. Geophys. Res. Lett. 2006, 33, 1–4. 10.1029/2005GL025629.

[ref11] Peterson K.; Cole-Dai J.; Brandis D.; Cox T.; Splett S. Rapid measurement of perchlorate in polar ice cores down to sub-ng L–1 levels without pre-concentration. Anal. Bioanal. Chem. 2015, 407, 7965–7972. 10.1007/s00216-015-8965-y. 26297465

[ref12] Angelis M. D.; Traversi R.; Udisti R. Long-Term Trends of Mono-Carboxylic Acids in Antarctica: Comparison of Changes in Sources and Transport Processes at the Two EPICA Deep Drilling Sites. Tellus, Ser. B: Chem. Phys. Meteorol. 2012, 64, 17331 10.3402/tellusb.v64i0.17331.

[ref13] Pokhrel A.; Kawamura K.; Seki O.; Matoba S.; Shiraiwa T. Ice core profiles of saturated fatty acids (C 12:0 -C 30:0 ) and oleic acid (C 18:1 ) from southern Alaska since 1734 AD: A link to climate change in the Northern Hemisphere. Atmos. Environ. 2015, 100, 202–209. 10.1016/j.atmosenv.2014.11.007.

[ref14] King A. C. F.; Thomas E. R.; Pedro J. B.; Markle B.; Potocki M.; Jackson S. L.; Wolff E.; Kalberer M. Organic Compounds in a Sub-Antarctic Ice Core: A Potential Suite of Sea Ice Markers. Geophys. Res. Lett. 2019, 46, 9930–9939. 10.1029/2019GL084249. 31762520 PMC6853201

[ref15] Battistel D.; Kehrwald N. M.; Zennaro P.; Pellegrino G.; Barbaro E.; Zangrando R.; Pedeli X. X.; Varin C.; Spolaor A.; Vallelonga P. T.; Gambaro A.; Barbante C. High-latitude Southern Hemisphere fire history during the mid- to late Holocene (6000-750 BP). Clim. Past 2018, 14, 871–886. 10.5194/cp-14-871-2018.

[ref16] Gambaro A.; Zangrando R.; Gabrielli P.; Barbante C.; Cescon P. Direct Determination of Levoglucosan at the Picogram per Milliliter Level in Antarctic Ice by High-Performance Liquid Chromatography/Electrospray Ionization Triple Quadrupole Mass Spectrometry. Anal. Chem. 2008, 80, 1649–1655. 10.1021/ac701655x. 18247516

[ref17] Grieman M. M.; Aydin M.; McConnell J. R.; Saltzman E. S. Burning-Derived Vanillic Acid in an Arctic Ice Core from Tunu, Northeastern Greenland. Clim. Past 2018, 14, 1625–1637. 10.5194/cp-14-1625-2018.

[ref18] Grieman M. M.; Aydin M.; Fritzsche D.; McConnell J. R.; Opel T.; Sigl M.; Saltzman E. S. Aromatic Acids in a Eurasian Arctic Ice Core: A 2600-Year Proxy Record of Biomass Burning. Clim. Past 2017, 13, 395–410. 10.5194/cp-13-395-2017.

[ref19] Grieman M. M.; Aydin M.; Isaksson E.; Schwikowski M.; Saltzman E. S. Aromatic Acids in an Arctic Ice Core from Svalbard: A Proxy Record of Biomass Burning. Clim. Past 2018, 14, 637–651. 10.5194/cp-14-637-2018.

[ref20] Zennaro P.; Kehrwald N.; McConnell J. R.; Schüpbach S.; Maselli O. J.; Marlon J.; Vallelonga P.; Leuenberger D.; Zangrando R.; Spolaor A.; Borrotti M.; Barbaro E.; Gambaro A.; Barbante C. Fire in Ice: Two Millennia of Boreal Forest Fire History from the Greenland NEEM Ice Core. Clim. Past 2014, 10, 1905–1924. 10.5194/cp-10-1905-2014.

[ref21] Oros D. R.; Simoneit B. R. T. Identification and Emission Factors of Molecular Tracers in Organic Aerosols from Biomass Burning Part 2. Deciduous Trees. Appl. Geochem. 2001, 16, 1545–1565. 10.1016/S0883-2927(01)00022-1.

[ref22] Oros D. R.; Simoneit B. R. T. Identification of Molecular Tracers in Organic Aerosols from Temperate Climate Vegetation Subjected to Biomass Burning. Aerosol Sci. Technol. 1999, 31, 433–445. 10.1080/027868299303986.

[ref23] Oros D. R.; Simoneit B. R. T. Identification and Emission Factors of Molecular Tracers in Organic Aerosols from Biomass Burning Part 1. Temperate Climate Conifers. Appl. Geochem. 2001, 16, 1513–1544. 10.1016/S0883-2927(01)00021-X.

[ref24] Fischer H.; et al. Reconstruction of Millennial Changes in Dust Emission, Transport and Regional Sea Ice Coverage Using the Deep EPICA Ice Cores from the Atlantic and Indian Ocean Sector of Antarctica. Earth Planet. Sci. Lett. 2007, 260, 340–354. 10.1016/j.epsl.2007.06.014.

[ref25] Candelone J.-P.; Hong S.; F. Boutron C. An Improved Method for Decontaminating Polar Snow or Ice Cores for Heavy Metal Analysis. Anal. Chim. Acta 1994, 299, 9–16. 10.1016/0003-2670(94)00327-0.

[ref26] Sigg A.; Fuhrer K.; Anklin M.; Staffelbach T.; Zurmuehle D. A Continuous Analysis Technique for Trace Species in Ice Cores. Environ. Sci. Technol. 1994, 28, 204–209. 10.1021/es00051a004. 22176163

[ref27] Spolaor A.; Vallelonga P.; Gabrieli J.; Roman M.; Barbante C. Continuous Flow Analysis Method for Determination of Soluble Iron and Aluminium in Ice Cores. Anal. Bioanal. Chem. 2013, 405, 767–774. 10.1007/s00216-012-6166-5. 22692592

[ref28] Burgay F.; Erhardt T.; Lunga D. D.; Jensen C. M.; Spolaor A.; Vallelonga P.; Fischer H.; Barbante C. Fe2+ in ice cores as a new potential proxy to detect past volcanic eruptions. Sci. Total Environ. 2019, 654, 1110–1117. 10.1016/j.scitotenv.2018.11.075. 30841386

[ref29] Federer U.; Kaufmann P. R.; Hutterli M. A.; Schüpbach S.; Stocker T. F. Continuous Flow Analysis of Total Organic Carbon in Polar Ice Cores. Environ. Sci. Technol. 2008, 42, 8039–8043. 10.1021/es801244e. 19031899

[ref30] Traversi R.; Becagli S.; Castellano E.; Maggi V.; Morganti A.; Severi M.; Udisti R. Ultra-Sensitive Flow Injection Analysis (FIA) Determination of Calcium in Ice Cores at Ppt Level. Anal. Chim. Acta 2007, 594, 219–225. 10.1016/j.aca.2007.05.022. 17586118

[ref31] Knüsel S.; Piguet D. E.; Schwikowski M.; Gäggeler H. W. Accuracy of Continuous Ice-Core Trace-Element Analysis by Inductively Coupled Plasma Sector Field Mass Spectrometry. Environ. Sci. Technol. 2003, 37, 2267–2273. 10.1021/es026452o. 12785535

[ref32] McConnell J. R.; Lamorey G. W.; Lambert S. W.; Taylor K. C. Continuous Ice-Core Chemical Analyses Using Inductively Coupled Plasma Mass Spectrometry. Environ. Sci. Technol. 2002, 36, 7–11. 10.1021/es011088z. 11811493

[ref33] Traversi R.; Becagli S.; Castellano E.; Migliori A.; Severi M.; Udisti R. High-Resolution Fast Ion Chromatography (FIC) Measurements of Chloride, Nitrate and Sulphate along the EPICA Dome C Ice Core. Ann. Glaciol. 2002, 35, 291–298. 10.3189/172756402781816564.

[ref34] Cole-Dai J.; Budner D. M.; Ferris D. G. High Speed, High Resolution, and Continuous Chemical Analysis of Ice Cores Using a Melter and Ion Chromatography. Environ. Sci. Technol. 2006, 40, 6764–6769. 10.1021/es061188a. 17144308

[ref35] Severi M.; Becagli S.; Frosini D.; Marconi M.; Traversi R.; Udisti R. A Novel Fast Ion Chromatographic Method for the Analysis of Fluoride in Antarctic Snow and Ice. Environ. Sci. Technol. 2014, 48, 1795–1802. 10.1021/es404126z. 24397469

[ref36] Severi M.; Becagli S.; Traversi R.; Udisti R. Recovering Paleo-Records from Antarctic Ice-Cores by Coupling a Continuous Melting Device and Fast Ion Chromatography. Anal. Chem. 2015, 87, 11441–11447. 10.1021/acs.analchem.5b02961. 26494022

[ref37] Grieman M. M.; Hoffmann H. M.; Humby J. D.; Mulvaney R.; Nehrbass-Ahles C.; Rix J.; Thomas E. R.; Tuckwell R.; Wolff E. W. Continuous Flow Analysis Methods for Sodium, Magnesium and Calcium Detection in the Skytrain Ice Core. J. Glaciol. 2021, 68, 90–100. 10.1017/jog.2021.75.

[ref38] Rubino M.; D’Onofrio A.; Seki O.; Bendle J. A. Ice-Core Records of Biomass Burning. Anthr. Rev. 2016, 3, 140–162. 10.1177/2053019615605117.

[ref39] Kuo L.-J.; Louchouarn P.; Herbert B. E. Influence of Combustion Conditions on Yields of Solvent-Extractable Anhydrosugars and Lignin Phenols in Chars: Implications for Characterizations of Biomass Combustion Residues. Chemosphere 2011, 85, 797–805. 10.1016/j.chemosphere.2011.06.074. 21762951

[ref40] Oros D. R.; Abas M. R. b.; Omar N. Y. M. J.; Rahman N. A.; Simoneit B. R. T. Identification and Emission Factors of Molecular Tracers in Organic Aerosols from Biomass Burning: Part 3. Grasses. Appl. Geochem. 2006, 21, 919–940. 10.1016/j.apgeochem.2006.01.008.

[ref41] Pápai Z.; Pap T. L. Analysis of Peak Asymmetry in Chromatography. J. Chromatogr. A 2002, 953, 31–38. 10.1016/s0021-9673(02)00121-8. 12058945

[ref42] Bliesner D. M. Validating Chromatographic Methods A Practical Guide; John Wiley & Sons, Inc.: Hoboken, New Jersey, 2006.

[ref43] Grieman M. M.; Greaves J.; Saltzman E. S. A Method for Analysis of Vanillic Acid in Polar Ice Cores. Clim. Past 2015, 11, 227–232. 10.5194/cp-11-227-2015.

[ref44] Zangrando R.; Barbaro E.; Vecchiato M.; Kehrwald N. M.; Barbante C.; Gambaro A. Levoglucosan and Phenols in Antarctic Marine, Coastal and Plateau Aerosols. Sci. Total Environ. 2016, 544, 606–616. 10.1016/j.scitotenv.2015.11.166. 26674690

[ref45] Zangrando R.; Barbaro E.; Zennaro P.; Rossi S.; Kehrwald N. M.; Gabrieli J.; Barbante C.; Gambaro A. Molecular Markers of Biomass Burning in Arctic Aerosols. Environ. Sci. Technol. 2013, 47, 8565–8574. 10.1021/es400125r. 23808421

[ref46] Matuszewski B. K.; Constanzer M. L.; Chavez-Eng C. M. Strategies for the Assessment of Matrix Effect in Quantitative Bioanalytical Methods Based on HPLC–MS/MS. Anal. Chem. 2003, 75, 3019–3030. 10.1021/ac020361s. 12964746

[ref47] Barbaro E.; Morabito E.; Gregoris E.; Feltracco M.; Gabrieli J.; Vardè M.; Cairns W. R. L.; Dallo F.; De Blasi F.; Zangrando R.; Barbante C.; Gambaro A. Col Margherita Observatory: A Background Site in the Eastern Italian Alps for Investigating the Chemical Composition of Atmospheric Aerosols. Atmos. Environ. 2020, 221, 117071 10.1016/j.atmosenv.2019.117071.

