# Supplementary material for: Fast Liquid Chromatography Coupled with Tandem Mass Spectrometry for the Analysis of Vanillic and Syringic Acids in Ice Cores
Source: Anal Chem. 2022 Mar 23;94(13):5344–51. doi: 10.1021/acs.analchem.1c05412 (PMC8988124; doi:10.1021/acs.analchem.1c05412)
Supplement: Supplementary file 1 — ac1c05412_si_001.pdf [file ac1c05412_si_001.pdf]

SUPPORTING INFORMATION

Fast liquid chromatography coupled with tandem mass spectrometry for analysis of vanillic and syringic acids in ice cores

Elena Barbaro[1,2], Matteo Feltracco[1,2]*, Azzurra Spagnesi[2], Federico Dallo[2,3], Jacopo Gabrieli[1,2], Fabrizio De Blasi[1,2], Daniele Zannoni[4,2], Warren R.L. Cairns[1,2], Andrea Gambaro[2,1], Carlo Barbante[1,2]

[1] Institute of Polar Sciences, National Research Council (CNR-ISP), Via Torino, 155 - 30172 Venice Mestre (VE), Italy
[2] Department of Environmental Sciences, Informatics and Statistics, Ca' Foscari University of Venice, Via Torino, 155 - 30172 Venice Mestre (VE), Italy
[3] Center for the Built Environment, University of California, 390 Wurster Hall #1839, Berkeley, CA 94720-1839
[4] Geophysical Institute, University of Bergen and Bjerknes Centre for Climate Research, Postboks 7803, NO-5020 Bergen, Norway

E-mail corresponding author: matteo.feltracco@unive.it

Table of contents

Details of Continuous Flow Analysis systems

Figure S1. Schematic of the continuous flow analysis (CFA) system optimized for coupling with the fast liquid chromatography- mass spectrometer (FLC-MS/MS).

Table S1. Description of the peristaltic pumps and tubing used in the CFA system.

Table S2. Optimized mass spectrometric parameters. DP: Declustering Potential, EP: Entrance Potential, CE: Collision Energy, CXP: Cell Exit Potential

Table S3. Chromatographic parameters used to evaluate the instrumental performance using a standard solution of vanillic (VA) and syringic (SyA) acids at a concentration of 100 pg mL^{-1} and the internal standard labelled vanillin (VAH*) at a concentration of 1 ng mL-1. Average values of peak area (in counts), peak width (in seconds), peak asymmetry and MS identification points for the first (C1) column (n=5) and the second (C2) column (n=5) and total average values together with the standard deviation in brackets are reported.

Details of the Continuous Flow Analysis system

The melting system is comprised of an aluminum anodized square-based melting head (external dimension of 32x32 mm^2 and internal dimension of 21x21 mm^2), specifically chosen after previous tests that were conducted with different melt head designs. The characteristic high walls separate the outer from the inner sections reducing the possibility of meltwater crossover, and any contamination potentially coming from the outermost melt water flow. Furthermore, the anodized aluminum guarantees chemical stability as well as having excellent thermal properties at a reasonable cost. The melting unit is thermostated between 30° and 33°C by an electrical heater coupled to a temperature PT100 sensor, located in an upright freezer kept at −20 °C. High-purity perfluoroalkoxy (PFA) tubing (1/16 in. OD, 0.02 in. ID) and standard 1/4–28 low-pressure fittings (both from Upchurch, USA) were used throughout the system. Ice was melted at a constant rate of 3 cm min^{-1} adjusting the temperature of the melt-head with a PID controller. The ice core samples have a square cross section as we receive a cut sub sample of the ice core. The stick is loaded into acrylic sample guides that are loaded onto the melter head that keep the sample upright. A weight is added to the top of the core to apply pressure to ensure smooth melting of the sample.

The melting line passes through a 10-port low-pressure switching valve (Cheminert, Vici) to allow rapid switching 1) to the sample line during ice core analysis, or 2) to ultra-pure water, during the wash phase. The switching valve was also used to load calibration standards before and after ice core analysis. Table S1 describes the overall CFA pumping system (peristaltic pumps and tubing). A first peristaltic pump (Ismatec ISM942) is set at 3.4 mL min^{-1} to 1) draw the sample from the melt-head, and 2) to remove external contaminated meltwater coming from the external surfaces of the ice core.

After an enclosed debubbler (c. 200 µL internal volume), for removing air bubbles from the liquid sample stream, the flow is split into two lines. The first line with a flow rate of 1.8 mL min^{-1} was collected by a fraction collector to obtain discrete samples. The second line with a flow rate of 1.6 mL min^{-1} brought the sample stream into a manifold. Here, the flow is split in three lines that are redirected to the same second peristaltic pump. In the first line, the insoluble particle counts (Abakus Klotz, Germany) and electrolytic conductivity (AmberScience, USA) are continuously determined online at a flow rate of 1.3 mL min^{-1} (green line in the Fig. S1). The second line (red in the Fig. S1) is directed to the injection system of the FLC-MS/MS to ensure a constant flow of 0.26 mL min^{-1}. A third line (blue stopper in the Fig. S1) is available for other analyses but is not used in this study. The use of fluoroelastomer (FPM) tubing in the FLC-MS/MS line is necessary because serious contamination problems were found using other peristaltic tubing materials (i.e. Tygon, Solvaflex, Marprene) when determining vanillic and syringic acids.

To guarantee a completely bubble-free stream to the chromatographic system, the sample passed through another debubbler (c. 100 µL internal volume) before being redirected again to the second peristaltic pump. This is because the ice can get stuck in the sample holder during the melting resulting in the introduction of air into the sample stream. There is switching valve, that allows for a quick change between sample to ultrapure water (UPW) to prevent large air bubbles being introduced, but despite these precautions bubbles can still enter the system. Therefore, the benefits offered by the second debubbler were considered higher than any potential contamination risk because the FLC system needs to be protected from air bubbles. The debubblers were placed under a small laminar flow hood to reduce potential contamination from the lab atmosphere. From our preliminary tests, no significant contamination was observed.

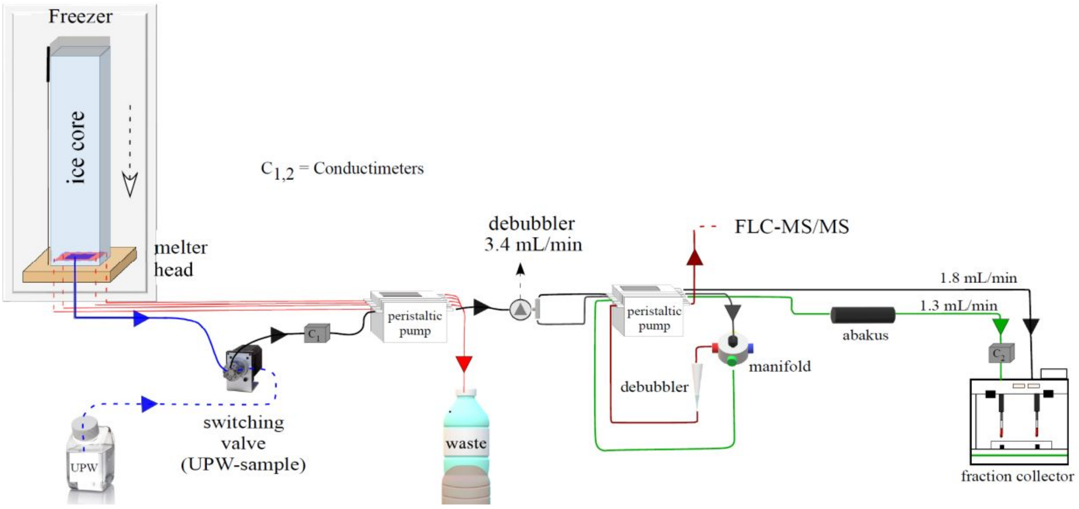

Figure S1. Schematic of the continuous flow analysis (CFA) system optimized for coupling with the fast liquid chromatography- mass spectrometer (FLC-MS/MS).

Table S1. Description of peristaltic pumps and tubing using in the CFA system.

<table>
<tr><th>Peristaltic pumps</th><th>model</th><th>tubing</th><th colspan="2">Used to:</th></tr>
<tr><td rowspan="2">1</td><td rowspan="2">Ismatec ISM942</td><td>1.02 mm ID fluoroelastomer (FPM) tubing (Agilent Technologies, Inc.)</td><td colspan="2">Connect the second peristaltic pump</td></tr>
<tr><td>2.29 mm ID Tygon tubing (Ismatech)</td><td colspan="2">remove external contaminated meltwater coming from the core surfaces.</td></tr>
<tr><td rowspan="5">2</td><td rowspan="5">Ismatech, ISM934C</td><td>1.09 mm ID Tygon tube (Ismatech)</td><td colspan="2">obtain discrete samples</td></tr>
<tr><td>1.02 mm ID FPM tubing line (Agilent Technologies, Inc.)</td><td colspan="2">brought into a manifold and the flow returns to this peristaltic pump as follows:</td></tr>
<tr><td rowspan="3"></td><td>Green line with Tygon tube (0.89 mm ID)</td><td>To determine online insoluble particle counts and electrolytic conductivity</td></tr>
<tr><td>Red line with a FPM tubing (0.38 mm ID)</td><td>To direct the sample stream into the injection system of the FLC-MS/MS</td></tr>
<tr><td>Blue stopper</td><td>is available for other analyses, and is not used in this work</td></tr>
<tr><td>3</td><td>Ismatech, ISM931C</td><td>0.25 mm ID Tygon tubing (Ismatech)</td><td colspan="2">To add internal standard into the FLC-MS/MS system</td></tr>
</table>

Table S2. Optimized mass spectrometric parameters. DP: Declustering Potential, EP: Entrance Potential, CE: Collision Energy, CXP: Cell Exit Potential

**Compound**	**Acronym**	**Q1 [M-H]$^-$ (m/z)**	**Q3 (m/z)**	**DP (V)**	**EP (V)**	**CE (V)**	**CXP (V)**	**CAD psi**
Vanillic acid	VA	166.9	**151.9**	-51	-10	-19	-10	6
			107.8	-51	-10	-28	-9	6
Syringic acid	SyA	196.8	**181.8**	-47	-10	-16	-15	6
			121.0	-47	-10	-23	-8.5	6
$^{13}C_6$- Vanillin	VAH*	156.9	141.8	-54	-10	-19	-8	6

Table S3. Chromatographic parameters used to evaluate the instrumental performance using a standard solution of vanillic (VA) and syringic (SyA) acids at a concentration of 100 pg mL^{-1} and internal standard labelled vanillin (VAH*) at a concentration of 1 ng mL^{-1}. Average values of peak area (in counts), peak width (in seconds), peak asymmetry and MS identification points for the first (C1) column (n=5) and the second (C2) column (n=5) and total average values. In the brackets the standard deviation is reported.

n-C1=5, n-C2=5, n=10	**VA**	**SyA**	**VAH***
peak area - C1 (counts)	7644(675) RSD% 9	7868(501) RSD% 6	458000(9626) RSD% 2
peak area - C2 (counts)	6992(460) RSD% 7	7416(505) RSD% 7	452500(14154) RSD% 3
peak area (counts)	*7318(644) RSD% 9*	*7642(531) RSD% 7*	*455250(11585) RSD% 3*
peak width – C1 (seconds)	4.1(0.3)	4.1(0.5)	5.1(0.2)
peak width – C2 (seconds)	4.1(0.6)	3.7(0.6)	5.4(0.5)
peak width (seconds)	*4.1(0.5)*	*3.9(0.5)*	*5.3(0.4)*
peak asymmetry – C1	1.6(0.2)	1.6(0.2)	2.2(0.4)
peak asymmetry – C2	1.7(0.2)	1.4(0.3)	2.8(0.4)
peak asymmetry	*1.6(0.2)*	*1.5(0.2)*	*2.5(0.5)*
MS identification points - C1	15(1)	14(2)	14(2)
MS identification points - C2	15(1)	14(1)	15(1)
MS identification points	*15(1)*	*14(1)*	*14(2)*
